# Review of the Literature: Organ of Giraldes Epididymal Appendage Presenting as a Painless Scrotal Mass in a 19-Year-Old Male—A Rare Urologic Entity

**DOI:** 10.1155/2015/748097

**Published:** 2015-10-21

**Authors:** Mohamad-Fadi Dalati, Tania Oliveira-e-Silva, Kim Entezari

**Affiliations:** ^1^CHU Saint-Pierre, 1000 Brussels, Belgium; ^2^Department of Urology, CHU Saint-Pierre, 1000 Brussels, Belgium

## Abstract

An incidental finding of a testicular mass in young male population is always a case of great concern for the patient and controversy for the physician. Differential diagnosis ranges from acute scrotum (notably testicular torsion), to acute inflammation and infection, all the way to testicular tumors. We present a case of an incidental finding of a painless testicular solid mass in a 19-year-old male patient, with an end pathological result of paradidymis (organ of Giraldes) following orchiectomy. To the best of our knowledge, this is the first case of its kind to be reported in the literature.

## 1. Introduction

**Figure 1:**
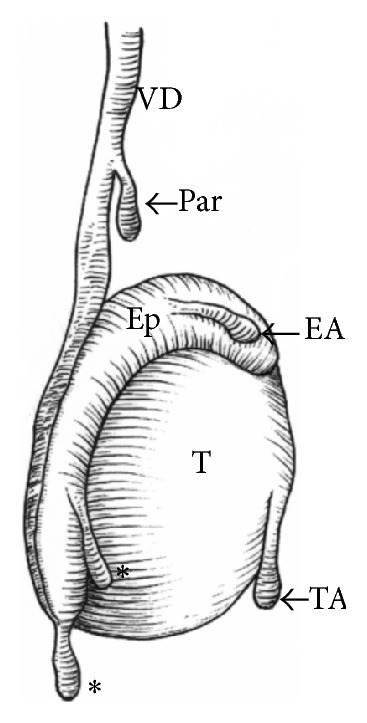
Testicular appendages; VD: vas deferens; T: testicle; Ep: epididymis; Par: organ of Giraldes; EA: epididymal appendage; TA: testicular appendage; *∗*: aberrans of Haller. Source: Favorito et al. [2].

A testicular appendage is a vestigial residual of the Wolffian (mesonephric) duct or the Mullerian (paramesonephric) duct. A Mullerian-inhibiting substance produced during fetal growth causes the degradation of the Mullerian duct, in a craniocaudal fashion [1]. There are 5 testicular/epididymal appendages described in literature.  Figure 1 illustrates the anatomical position of these appendages [2]. The first and the most cranial part develops into the appendix testis, also known as the sessile hydatid of Morgagni. Next in order originating from the head of the epididymis is the appendix epididymis. The third appendage is the paradidymis, also known as the organ of Giraldes (Par), which attaches to the lower spermatic cord, and is gaining an origin from the Wolffian (mesonephric) duct, mainly from its caudal portion. Originating from the body of the epididymis is the cranial aberrant duct, also called the cranial vas aberrans of Haller (*∗*), which also originated from the Wolffian duct. Finally at the level of the tail of the epididymis, we have the caudal vas aberrans of Haller (*∗*). Pathologies and cases involving these appendages are quite rare and almost always involve a presentation of an acute scrotal mass.

## 2. Case

We describe a case of a healthy 19-year-old male patient, presenting to the urology clinic for an incidental finding of a painless left scrotal mass. Clinical history goes back to a couple of days before, where the patient first noticed a mass while taking a shower. The patient denies recent scrotal trauma, unprotected sexual intercourse, penile discharge, urinary symptoms, fever, or chills. Physical exam revealed a 3-4 cm scrotal mass, attached to the left testicle at the level of the epididymis. The mass was painless to palpation and mobile with the testicle. No inguinal hernia or inguinal lymph nodes were detected during the physical exam. Both testicles were of normal size and position. Cremasteric reflex was present bilaterally. No signs of inflammation, edema, erythema, or infection were observed. Blood and urine exams were within normal ranges, including hemoglobin, Hb, hematocrit, Hct, white blood cells, WBC, C-reactive protein, CRP, negative red blood cells, RBC, and white blood cells, WBC, in urine and negative urine culture. Tumor markers (alpha fetoprotein, AFP, *β*-HCG, and lactate dehydrogenase, LDH) were also negative. Ultrasound of the scrotum paradoxically revealed a swelling of the left epididymis and the testicle with hypervascularization signals on Doppler ultrasound suggestive of epididymitis.

The decision was made to treat the subclinical, ultrasound-evident epididymitis with a course of fluoroquinolones (ciprofloxacin). Two weeks later, physical exam showed similar findings to the one done two weeks ago, and testicular ultrasound showed a 3 cm testicular swelling, with similar Doppler findings.

This atypical presentation of a painless scrotal mass in a young male adult, with negative tumor markers and ultrasound suggestive of epididymitis in the absence of any inflammatory signs or symptoms, with no signs of improvement with a course of antibiotics, as well as the risk of malignant lesion, put a remarkable amount of stress on the patient and the treating team, resulting in a decision to go for surgical testicular exploration via an inguinal incision due to the risk of testicular malignancy. Metastatic workup was composed of fluorine-18-fluorodeoxyglucose positron emission tomography ^18^F-FDG PET-CT, showing a hypermetabolic lesion of the left testicle, with iliac and para-aortic lymphadenopathy.

During surgery, the left testicle was delivered via an inguinal incision. Dissection of the mass off the testicle was tried, but due to the adherent nature of the mass to the testicle itself, increasing the risk of malignancy, the final decision was made to undergo a total left radical orchiectomy. Patient was discharged the following day.

**Figure 2:**
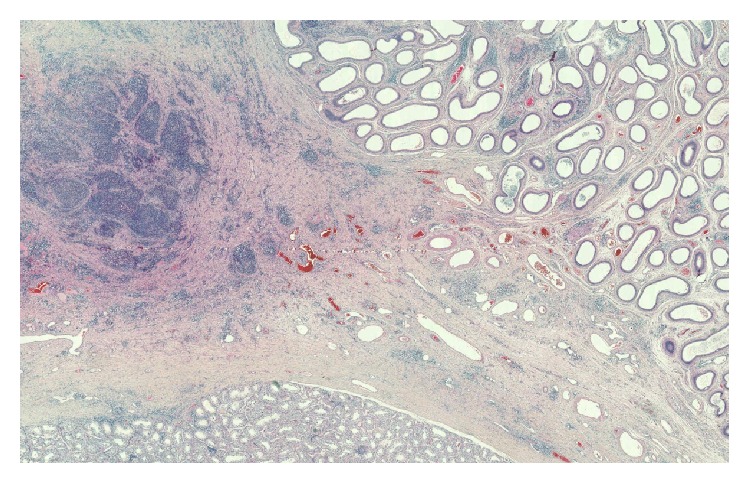
Testicular parenchyma in the bottom, epididymis in the upper right, and inflammatory vestigial remnants in upper left.

**Figure 3:**
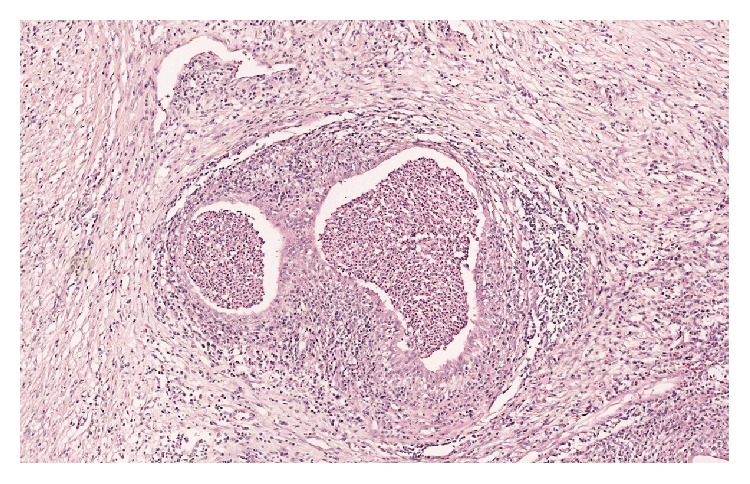
Microabscess in vestigial remnants.

Pathology report showed a normal testicle (6.5 × 4 × 3.7 cm), spermatic cord, and an epididymis containing an indurated whitish lesion, measuring 3.5 × 2 × 1.6 cm. No histological anomalies of the testicle were noted. A significant inflammatory remnant of the epididymis was reported, with microabscess in vestigial remnants (Figures 2 and 3).

A cystic structure with no obvious continuity with the epididymis was also noted, bordered by a pseudostratified epithelium without tumoral cellular atypia, consistent with organ of Giraldes, with no sign of malignancy.

^18^F-FDG PET-CT was repeated 2 months after surgery and showed complete remission of the previously hypermetabolic picture that was reported in the previous imaging.

## 3. Discussion

In a study by Sahni et al. [3], the incidence of epididymal appendage in adults on autopsy was estimated to be around 20%. In another study by Favorito et al. [2], the incidence of epididymal appendages was 14.5% in the cryptorchidism group and 8.4% in the control one, with no statistically significant difference between the two groups. The organ of Giraldes as a cause of a scrotal mass is a very rare pathologic finding, with cystic transformation and torsion being the most common presentation. It is usually located in the anteroinferior portion of the spermatic cord, varying in size, with no direct relationship to the epididymis or the testicle. Diagnosis is very challenging, almost always done only after surgical excision. Ultrasound of the scrotum and Doppler ultrasound for assessment of testicular perfusion fail to confirm the diagnosis and are often inconclusive.

There are no studies indicating risk of malignancy of these appendages, nor there were, to the best of our knowledge, any case reports of a painless scrotal mass, which was surgically explored, turning out to be an appendage.

Torsion of the appendages, mainly in adolescents, remains to be a risk to consider. Van Glabeke et al. described 543 cases of acute scrotum pain in boys aged between 1 and 16 years, resulting in surgical exploration [4], of which 46% were due to torsion of appendages. Puri and Boyd [5] stated that torsion of testicular/epididymal appendages is very rare after the age of 20 years because of what they referred to as “local fibroses.” Their study reported 22 cases of torsion of testicular/epididymal appendages. In a retrospective study done by Khairi et al. [6], and over a period of 7 years, 34 cases of acute scrotum were managed by surgical exploration, of which 7 cases (20.5%) were testicular/epididymal appendages torsion. In Turkey, a study by Çavuşoglu et al. [7], 32.3% of cases of acute scrotum, managed by surgical exploration, were testicular/epididymal appendages.  Table 1 summarizes these findings.

While the usage of ^18^F-FDG PET-CT is controversial in testicular cancer diagnosis according to the guidelines of the European Association of Urology, there are many studies in the literature favoring its usage. A meta-analysis done by Zhao et al. [8] examined a total of 16 studies, with a total number of patients up to 807 and 957 ^18^F-FDG PET exams. The meta-analysis showed sensitivity of 87% and specificity of 75%. It concluded that combining CT with FDG-PET is potentially a useful tool in diagnosis of testicular cancer, while admitting the low specificity of such imaging technique. In our hospital, we use ^18^F-FDG PET-CT in the metastatic workup before surgical exploration, as well as during follow-up cycles.

Even though surgical excision is theoretically not mandatory in case of testicular and epididymal appendages, due to the benign nature of these structures, with no signs of torsion, the burden of a solid scrotal mass in young males associated with the risk of testicular malignancy, even in the absence of elevated tumor markers or suspicious features of ultrasound, challenges both patient and urologist and drives management towards surgical exploration and excision.

In our opinion, the classic approach to scrotal masses in young male population, including physical exam, scrotal ultrasound, and tumor markers (AFP, *β*-HGC, and LDH), cannot give a clear contribution for the diagnosis of organ of Giraldes, leaving surgical exploration and eventually orchiectomy a must-do approach. Our case represents a rare entity in the literature, of a solid scrotal mass, with no other associated symptoms, ending up with surgical exploration and orchiectomy for the suspicion of testicular cancer, with an end result of organ of Giraldes. We believe that solid masses, even in the absence of supporting diagnostic measures, should be managed by surgical exploration and confirmation of absence of malignancy by pathology.

## Figures and Tables

**Table 1:** Summary of studies describing testicular and epididymal appendages.

Study	Incidence of appendages	Incidence after surgical exploration (acute scrotum)
Sahni et al., 1996 [3]	20%	

Favorito et al., 2004 [2]	14.5% in cryptorchidism 8.4% in control	

Van Glabeke et al., 1999 [4]		46% torsion of appendages

Puri and Boyd, 1976 [5]		22 cases

Khairi et al., 2007 [6]		20.5% torsion of appendages

Çavuşoglu et al., 2005 [7]		32.3% torsion of appendages
